# Can adherence to moral standards and ethical behaviors help maintain a sense of purpose in life? Evidence from a longitudinal study of middle-aged and older adults

**DOI:** 10.1371/journal.pone.0273221

**Published:** 2022-08-19

**Authors:** Dorota Weziak-Bialowolska, Piotr Bialowolski

**Affiliations:** 1 Centre for Evaluation and Analysis of Public Policies, Faculty of Philosophy, Jagiellonian University, Cracow, Poland; 2 Human Flourishing Program, Institute for Quantitative Social Science, Harvard University, Cambridge, MA, United States of America; 3 Department of Economics, Kozminski University, Warsaw, Poland; University Hospital Marques de Valdecilla, SPAIN

## Abstract

Personal factors, such as character strengths, have been shown to be favorably associated with concurrent and future well-being. Positive associations have also been reported between purpose in life and concurrent and subsequent health and well-being. Evidence on antecedents of purpose in life is, however, limited. This study examines whether the adherence to moral standards and ethical behaviors (AMSEB) is associated with subsequent purpose in life. Data from the Health and Retirement Study obtained from a sample of 8,788 middle-aged and older adults in the US (mean age = 64.9 years, age range 50–96 years) were used. The prospective associations between AMSEB and purpose in life were examined using generalized linear models. A rich set of covariates and prior outcomes were used as controls to reduce the risk of reverse causation. The robustness analyses included computation of sensitivity measures, E-values, and running a set of secondary analyses conducted on subsamples of respondents and using a limited set of covariates. It was found that middle-aged and older adults who demonstrated higher AMSEB reported a higher sense of purpose in life after the 4-year follow-up period. This association was found to be monotonic, moderately robust to potential unmeasured confounding and independent of demographics, prior socioeconomic status, prior health conditions, and health behaviors as well as prior psychological predispositions such as dispositional optimism and life satisfaction. It was also robust to missing data patterns. Policymakers and health practitioners may consider a predisposition to adherence to moral standards and ethical behaviors as a potential intervention target, as its improvement and/or maintenance has the potential to improve longevity and to help promote healthy and purposeful aging.

## Introduction

A sense of purpose in life refers to an inclination to derive meaning from life events and to feel a sense of direction in life. It has also been perceived as a central component of eudaimonic well-being [1–3]. This trait-like tendency to pursue goals and have a sense of accomplishment has been recently recognized as a protective factor against ill health and as a positive health asset contributing to human flourishing. In particular, for example for middle-aged and older adults, there is accumulating observational and experimental evidence suggesting that demonstrating a higher sense of purpose and meaning is prospectively associated with good mental and physical health, more prevalent use of preventive care services, lower risk of unhealthy behaviors, a reduced risk of developing impairment in basic and instrumental activities of daily living as well as mobility limitations, lower risk of dementia onset and the pathologic changes of Alzheimer’s disease to cognition, fewer cardiovascular events such as myocardial infarction, lower risk of stroke, fewer sleep disturbances, and reduced mortality risk [4–15].

Although sense of purpose in life has been a well-recognized factor positively affecting health, well-being and longevity, especially in middle-aged and older adulthood, evidence on its antecedents is only emerging [16]. Some preliminary evidence from the population of US nurses, US students, Mexican factory workers and UK middle-aged and older adults suggests that positive affect, social connections and feeling of purpose while at work are associated with subsequent higher levels of purpose and meaning in life, while psychological distress is associated with subsequent lower levels of purpose and meaning in life [17–20]. Additionally, for middle-aged and older adults in the UK, it has been reported that favorable economic conditions and greater physical activity can positively contribute to an improved sense of a meaningful life at a 2-year follow-up [18]. Regarding physical health, for middle-aged and older adults from the UK, it has been reported that prior number of chronic diseases and prior lack of pain are associated with a greater experience of meaning in life [18], but no prospective associations with purpose in life have been reported for prior health behaviors or prior physical health among US nurses [17].

Therefore, more research is still needed to better understand the determinants and mechanisms of promoting, maintaining, and restoring purpose in life as a health asset. These aspects are crucial to designing effective interventions with a sense of purpose as a resource for promoting health- and well-being and/or preventing disease.

Consequently, this study aims to examine adherence to moral standards and ethical behaviors as an antecedent to purpose in life. Our interest in this particular exposure results from, first, the previous arguments that finding purpose in life may be consequential of personal attributes related to personality traits [21, 22] and associated with prior levels of orientation to do and promote good [23]. Second, it is ensuing from theoretical arguments that morally valued personality traits that are fundamental to one’s identity produce positive outcomes for oneself and/or others, and contribute to the greater good [24, 25], were categorized as personal enablers of well-being and mitigators against the unfavorable impact of life events and difficulties [3, 26]. Third, our interest also results from prior theoretical arguments and empirical findings indicating that the predisposition to act according to ethical standards and accepted rules of good, honest and/or moral behaviors, as well as having thoughts and taking actions that contribute to the good of oneself and others, contribute to an attainment of complete eudaimonic well-being and better health [23, 27–30]. In particular, prior research indicate that a tendency to act according to moral standards and ethical behaviors is associated with lower risks of incident cognitive impairment not dementia, depression and unfavorable health-related behaviors as well as lower limitations in mobility and less difficulty in instrumental activities of daily living among middle-aged and older adults [30–32]. Additionally, other studies on related constructs have also shown that prosocial behaviors such as generosity or kindness, providing emotional and economical support to others, and performing acts of altruistic behaviors may favorably affect health and increase the well-being of the giver [33–36]. Consequently, in this study, we test the hypothesis that the adherence to moral standards and ethical behaviors is favorably associated with higher subsequent purpose in life, and this prospective association holds even after adjusting for a wide range of potential confounders (i.e., sociodemographic and psychological factors, health behaviors, and prior health conditions).

## Materials and methods

### Data

The main source of data was the nationally representative panel study–the Health and Retirement Study (HRS)–which is conducted every two years in the US and collects data from people aged 50 and older (henceforth referred to as middle-aged and older adults) [37, 38]. In this study, we used demographic, socioeconomic and health data from the HRS core questionnaire collected in 2004–2014 as well as psychological data collected using the ‘Psychosocial and Lifestyle Questionnaire’ in waves 2008/2010-2012/2014 (all waves in which these data were collected). In the construction of the analytical sample, we took into account the fact that the HRS uses a mixed-mode design for collecting data from the ‘Psychosocial and Lifestyle Questionnaire’, which implies that the alternating random 50% subsample of the longitudinal panel is asked to fill in this questionnaire every 4 years [38, 39]. Therefore, we combined data from two subcohorts (2008 and 2010) to increase the sample size and to improve statistical power. Consequently, three “combined” waves (i.e., pre-baseline, baseline and outcome waves, being 4 years apart from each other) were analyzed.

The analytical sample was limited to individuals who (i) completed the AMSEB questions and (i) purpose in life questions from the ‘Psychosocial and Lifestyle Questionnaire’ at baseline. This resulted in a final sample size of 8,788 middle-aged and older adults. Detailed information concerning methodological aspects of the HRS as well as access to data is available at the HRS website (https://hrs.isr.umich.edu/). Because this study used deidentified, publicly available data, it was exempted from review by the Harvard Longwood Campus Institutional Review Board.

### Measures

In this study, we used both single questions and psychosocial scales available in the HRS. They have been shown to be useful in previous studies, and the scale has also been rigorously validated [37, 39, 40].

#### Adherence to moral standards and ethical behaviors

Adherence to moral standards and ethical behaviors was assessed using a four-item instrument “Virtue of adherence to moral standards and ethical behavior” available in the HRS [39, 41]. The instrument constitutes a subscale of the conscientiousness measure and is conceptualized to capture beliefs corresponding to adherence to moral standards of honesty and as a predisposition to act in accordance with established rules of good and ethical behavior as well as attempting to be a moral exemplar [41]. Therefore, henceforth, this scale will be referred to as the scale of adherence to moral standards and ethical behaviors (AMSEB). The AMSEB items comprise: ‘If I could get away with it, I would not pay taxes’ (reverse scored); ‘I could be insincere and dishonest, if the situation required me to do so’ (reversed scored); ‘If the cashier forgot to charge me for an item, I would tell him or her’; ‘When I was in school, I would rather get a bad grade than copy someone else’s homework’. Agreeability with each item was assessed using a six-point Likert-type scale: 1 = Strongly disagree, 2 = Somewhat disagree, 3 = Slightly disagree, 4 = Slightly agree, 5 = Somewhat agree, 6 = Strongly agree. Scores for negatively worded items were reversed, and the scale of AMSEB was calculated as an average of the scores across items. Consequently, the scale ranges from 1 to 6, and higher score is indicative of higher adherence to moral standards and ethical behaviors reflected in a predisposition to act in accordance with established rules of good, honest, and ethical behavior. As suggested by the HRS guide [39], the final score was set to missing if there were more than two items with missing values.

The instrument has been psychometrically validated (including established unidimensionality, satisfactory reliability and validity) in the US and UK populations [41, 42]. It has also been used in various studies of mental and physical health, cognitive impairment, well-being, and quality of life [30, 31, 43].

The AMSEB was assessed in the baseline wave (2008/2010; the only waves in which this variable was measured). It was standardized (mean = 0, standard deviation = 1) and used in the analyses as a continuous variable. Additionally, to examine possible nonlinear threshold effects, a categorical variable according to the tertiles of data based on the baseline distribution of the AMSEB scale scores in the analytic sample was constructed and used in the secondary analyses.

#### Purpose in life

Purpose in life was assessed using a seven-item instrument available in the HRS. The instrument constitutes a subscale of the Psychological Well-Being measure [2]. It intends to capture the belief that one’s life is purposeful and meaningful. The instrument has been psychometrically validated [44] and proved useful in various studies on associations between sense of purpose in life and health outcomes [8, 45, 46]. The exemplary items include ‘I have a sense of direction and purpose in my life’ and ‘I do not have a good sense of what it is I’m trying to accomplish in life’. Respondents rated their agreeability with items using a six-point Likert-type scale: 1 = Strongly disagree, 2 = Somewhat disagree, 3 = Slightly disagree, 4 = Slightly agree, 5 = Somewhat agree, 6 = Strongly agree. Negatively worded items were reverse scored, and the scale of purpose in life was calculated as an average of the scores across all seven items. Following the HRS protocols [39], the final score was set to missing if there were more than three items with missing values. The scale ranged from 1 to 6, with higher scores indicating greater levels of purpose in life.

In the study, we considered the purpose in life a standardized continuous variable (mean = 0, standard deviation = 1). Following the study design, the purpose in life was assessed 4 years after the AMSEB exposure in the outcome wave (2012/2014). To reduce the risk of reverse causation, we also controlled for the prior level of purpose in life at baseline (the measurement of this variable started in HRS in 2006 and limited the possibility to control for it in the prebaseline wave).

#### Covariates

Prior research indicated a wide range of possible predictors of purpose in life and character strengths including demographic (e.g., gender, age, race/ethnicity, and marital status), economic (e.g., income and wealth), psychological (e.g., positive and negative affect), and health factors (e.g., health behaviors and health condition) [30, 47–51]. Therefore, we adjusted for participants’ characteristics including sociodemographic factors, health behaviors, health conditions and psychological factors. Regarding sociodemographic variables, we controlled for age (50–59, 60–69, 70–80, 80+), gender (male or female), race (White/Caucasian, Black/African American, other), educational attainment (less than high school, GED, high school graduate, some college, college and above), marital status (married, married but spouse absent, partnered, separated, divorced, widowed, never married), annual personal income (logarithm), and household wealth (logarithm). With respect to health behaviors, alcohol consumption (number of days per week), smoking (yes or no) and BMI were accounted for in the analyses. We also controlled for health conditions, including in the set of covariates self-reported variables on the presence/absence of obtaining a doctor’s diagnosis for six physical health conditions, such as stroke, diabetes, cancer, lung disease, heart condition, and high blood pressure. Next, a mental health condition was controlled for. Depression was measured by the Center for Epidemiological Studies Depression eight-item scale (CES-D8) [52]. Additionally, two physical functioning factors were also controlled for: limitations in instrumental activities of daily living as measured by the IADL index available in the RAND HRS Longitudinal File 2016 [53–55] and limitations in mobility, strength and fine motor skills reflected in the mobility index also already available in the RAND HRS Longitudinal File 2016 [55]. Finally, psychological predispositions such as life satisfaction measured with the 5-item Diener Satisfaction with Life Scale [56] and dispositional optimism assessed with the Life Orientation Test [57] were accounted for.

Sociodemographic covariates, health behaviors and physical and mental health conditions, including daily life functioning, were assessed via self-reports in the prebaseline wave (2004/2006). Psychological covariates were measured in the baseline wave. The assessment in the prebaseline wave was not feasible due to (1) the mixed-mode design with biennially alternating samples and (2) the fact that the ‘Psychosocial and Lifestyle Questionnaire’ was introduced in 2006 and administered only to alternating half of the sample [39].

### Statistical analysis

All statistical analyses were performed using Stata/SE 17.0 for Mac.

The prospective associations were modeled using generalized linear models. Two alternative specifications of the exposure variable were examined. The AMSEB scale was applied as a standardized continuous variable (mean = 0, standard deviation = 1) as well as a categorical variable indicating the tertiles of the AMSEB scale. The aim was to examine possible nonlinear threshold effects. Standardized regression estimates were reported. Control for prebaseline (if prebaseline was not available, the baseline was used instead) covariates, and prior outcome was used to reduce the risk of reverse causation.

Since we used a rich set of covariates, the risk of overfitting the model emerged. Therefore, the primary model was run under three alternative specifications, excluding particular subsets of covariates. In Model 1, we controlled only for social determinants of health, that is, sociodemographic characteristics, wealth, and income. In Model 2, compared to Model 1, we added variables related to health behaviors. Model 3 additionally controlled for health conditions, and Model 4 (i.e., the primary model) included all covariates, that is, additional psychosocial factors. To decrease the risk of reverse causation, all models also controlled for the prior outcome.

To examine the generalizability of the results, potential interactions between AMSEB and sociodemographic covariates (i.e., gender, age, education, race, income and total wealth) were examined. The moderating effects were tested in the model with the most extensive set of covariates (i.e., Model 4).

All missing covariate and outcome variables were imputed using the chained equations. Ten sets of imputed data were generated [58], and the multiple imputation estimates pooled using Rubin’s rule [59] were presented. A series of robustness checks was also conducted. First, the robustness of the results was examined using the E-values [60]. These are sensitivity measures that aim to examine the magnitude of association between a potential unmeasured confounder and both the exposure and the outcome to entirely cancel out the observed association. Second, all models were rerun after excluding anyone with a history of chronic conditions (i.e., heart attack, hypertension, high blood cholesterol, stroke, diabetes, cancer and depression; the threshold ≥3 on CES-D8 was used to classify respondents as having clinically significant depressive symptoms [52]). Finally, the primary set of models was reanalyzed using complete-case analysis to assess the robustness of the results to missing data patterns.

## Results

### Descriptive analysis

In the prebaseline wave (2004/2006), participants were 64.9 (SD = 8.47, age range 50–96 years) years old on average. They were mostly women (59.7%), married (64.1%), predominantly Caucasian (84.4%), and had a high school education (32.8%). They scored 5.0 on average on the AMSEB scale (SD = 0.95l range: 1–6) and were mostly healthy at baseline. Participants scoring higher on the AMSEB scale, compared with those scoring the lowest, reported more favorable health behaviors and better psychological conditions. In Table 1 we present participant characteristics by AMSEB tertiles.

**Table 1:** Distribution of participant characteristics by adherence to moral standards and ethical behaviors at study baseline, health and retirement study, US.

	AMSEB							
Participant Characteristic	Total (N = 8,788)		Tertile 1 (N = 3,620)		Tertile 2 (N = 2,870)		Tertile 3 (N = 2,298)	
	%	Mean (SD)	%	Mean (SD)	%	Mean (SD)	%	Mean (SD)
***Sociodemographic factors***								
Gender (male)	40.2		47.10		40.59		29.29	
Age group								
50–59	29.72		33.40		29.23		24.54	
60–69	40.76		40.39		41.43		40.51	
70–79	24.23		21.57		25.05		27.37	
80+	5.29		4.64		4.29		7. 57	
Race								
White/Caucasian	84.35		80.35		86.83		85.99	
Black/African American	11.27		13.12		9.62		10.40	
Other	4.38		5.52		3.55		3.61	
Marital status								
Married	64.14		63.43		66.03		62.91	
Married but spouse absent	0.66		0.64		0.59		0.78	
Partnered	3.03		3.90		2.75		2.00	
Separated	1.06		1.33		0.84		0.91	
Divorced	10.18		10.80		9.97		9.27	
Widowed	17.94		16.47		17.45		21.03	
Never married	3.00		3.45		2.37		3.09	
Education attainment								
Less than high school	15.49		18.71		11.12		15.88	
GED	4.61		5.06		4.25		4.35	
High school graduate	32.75		31.80		32.14		34.99	
Some college	23.51		22.13		24.05		25.02	
College and above	23.64		22.30		28.44		19.76	
Annual personal income ($)		17,068 (77,631)		19,044(113,67)		18,823 (39,353)		11,781 (26,898)
Household net financial assets ($)		441,405 (1,092,141)		404,816 (906,498)		492,671 (1,283,796)		435,166 (1,097,899)
***Health Behaviors***								
Alcohol consumption (no. of days per week)		1.22 (2.12)		1.33 (2.20)		1.28 (2.16)		0.95 (1.93)
Smoking (yes)	54.64		60.34		52.70		48.10	
BMI		28.25 (5.63)		28.61 (5.64)		28.02 (5.53)		27.97 (5.72)
***Psychosocial factors***								
Dispositional optimism; 1–6		4.55 (1.13)		4.37 (1.15)		4.61 (1.04)		4.78 (1.16)
Life satisfaction; 1–7		5.03 (1.49)		4.83 (1.50)		5.09 (1.42)		5.27 (1.52)
***Physical Health***								
Diagnosis of stroke	3.77		4.10		3.50		3.60	
Diagnosis of diabetes	16.12		17.96		14.99		14.64	
Diagnosis of cancer	12.15		11.23		12.95		12.59	
Prior diagnosis of chronic lung disease	7.86		8.33		7.39		7.71	
Diagnosis of heart condition	20.01		19.65		20.33		20.19	
Diagnosis of high blood pressure	52.67		53.56		51.65		52.52	
***Mental Health***								
Depression based on CES-D8 scale; 0–8		1.22 (1.84)		1.41 (1.95)		1.07 (1.70)		1.11 (1.79)
Depression based on CES-D8≥3	16.78		19.87		14.35		14.95	
***Daily functioning***								
Limitations with instrumental activities of daily living scale (IADL); 0–3		0.05 (0.26)		0.06 (0.29)		0.03 (0.22)		0.04 (0.25)
Mobility Index; 0–5		0.80 (1.21)		0.84 (1.23)		0.72 (1.17)		0.82 (1.23)

AMSEB = adherence to moral standards and ethical behavior, SD = standard deviation, BMI = body mass index.

### Adherence to moral standards and ethical behaviors and purpose in life

During the 4-year follow-up period, middle-aged and older adults who scored higher on the AMSEB scale had a substantially greater sense of purpose in life. These prospective associations were highly consistent across all four models (Table 2, Models 1–4). After adjusting for sociodemographic factors, prior health behaviors and prior health conditions (Model 3), one standard deviation increase in AMSEB was associated with a subsequent increase in the sense of purpose in life by 0.055 points on its standardized score (*β* = 0.055, 95% CI = 0.037; 0.074; p<0.001). Controlling for each additional set of covariates led to an attenuation of the prospective association between AMSEB and purpose in life. However, the association remained significant (p<0.001) in all of the models (Table 2, Models 1–4). Finally, potential interactions between AMSEB and gender, age, education, race, income, and total wealth were examined. The moderating effects were found not to be significant for any of the covariates (S1 Table).

**Table 2:** Standardized regression estimates for the association between baseline adherence to moral standards and ethical behaviors and purpose in life over a four-year follow-up period in middle-aged and older adulthood. Health and Retirement Study, US, 2012/2014–2016/2018, n = 8,497^b^.

Model	Standardized AMSEB		Tertile 1	Tertile 2		Tertile 3	
	*β* (95% CI)	p-value	Reference	*β* (95% CI)	p-value	*β* (95% CI)	p-value
Model 1^a^	0.064 (0.045; 0.082)	<0.001	Reference	0.063 (0.022; 0.104)	0.003	0.168 (0.123; 0.213)	<0.001
Model 2^a^	0.061 (0.043; 0.080)	<0.001	Reference	0.058 (0.016; 0.099)	0.006	0. 163 (0.117; 0.209)	<0.001
Model 3^a^	0.055 (0.037; 0.074)	<0.001	Reference	0.052 (0.011; 0.094)	0.013	0.156 (0.110; 0.201)	<0.001
Model 4^a^	0.046 (0.028; 0.064)	<0.001	Reference	0.040 (-0.001; 0.081)	0.053	0.134 (0.089; 0.179)	<0.001

AMSEB = adherence to moral standards and ethical behaviors; CI = confidence interval

^a^ Model 1 controls for sociodemographic characteristics, wealth, income + prior purpose in life; Model 2 is controls for sociodemographic characteristics, wealth, and income + health behaviors + prior purpose in life; Model 3 controls for sociodemographic characteristics, wealth, and income + health behaviors + health conditions + prior purpose in life; Model 4 controls for all covariates + prior purpose in life.

^b^ All missing covariate and outcome variables were imputed using the chained equations. Ten sets of imputed data were generated [58], and the multiple imputation estimates pooled using Rubin’s rule [59] are presented.

The analysis also confirmed a monotonic association between AMSEB and subsequent purpose in life (Table 2; columns–tertile 1, tertile 2 and tertile 3). Compared with individuals who scored the lowest on the AMSEB, participants who scored in the third tertile after the 4-year follow-up period had higher scores on the purpose in life scale (*β* = 0.134; 95% CI = 0.089; 0.179, p<0.001). Similar to the previous analyses, adding each additional set of covariates resulted in a slight attenuation of the prospective association between AMSEB and purpose in life.

### Robustness analysis

Sensitivity analyses conducted with the E-values showed that the observed association between AMSEB and purpose in life was moderately robust to potential unmeasured confounding (e.g., confounding by a personality factor such as neuroticism) (Table 3). For example, to explain away the observed prospective association between AMSEB and purpose in life, an unmeasured confounder would need to be associated with both AMSEB and purpose in life by risk ratios of 1.25 each above and beyond the measured covariates for the standardized AMSEB and of risk ratio of 1.51 each above and beyond the measured covariates for the third tertile of AMSEB. The weaker confounder would not be sufficient. Regarding the E-value for the limit of the 95% CI, to shift the lower limit of the CI for the observed association between AMSEB and purpose in life to include the null value, this unmeasured confounder would need to be associated with both AMSEB and purpose in life by 1.19-fold each, above and beyond the measured covariates for the standardized AMSEB and by 1.39-fold each, above and beyond the measured covariates for the third tertile of AMSEB.

**Table 3:** Robustness to unmeasured confounding (E-values) for assessing the associations between adherence to moral standards and ethical behaviors (standardized values and tertile 3 vs. tertile 1) and subsequent health outcomes in middle-aged and older adulthood, health and retirement study, US^d^.

	Standardized AMSEB		Tertile 3 vs. tertile 1	
	E-Value for Effect Estimate^a^	E-Value for CI Limit^b^	E-Value for Effect Estimate^a^	E-Value for CI Limit^b^
Model 1^c^	1.31	1.25	1.60	1.48
Model 2^c^	1.30	1.24	1.59	1.47
Model 3^c^	1.28	1.22	1.57	1.45
Model 4^c^	1.25	1.19	1.51	1.39

AMSEB = adherence to moral standards and ethical behaviors; CI = confidence interval.

^a^ The E-values for effect estimates are the minimum strength of association on the risk ratio scale that an unmeasured confounder would need to have with both the exposure and the outcome to fully explain away the observed associations of receiving employee recognition with various health and well-being outcomes, conditional on the measured covariates.

^b^ The E-values for the limit of the 95% CI closest to the null denote the minimum strength of association on the risk ratio scale that an unmeasured confounder would need to have with both the exposure and the outcome to shift the confidence interval to include the null value, conditional on the measured covariates.

^c^ Model 1 is run controlling for sociodemographic characteristics, wealth, income + prior purpose in life; model 2 is run controlling for sociodemographic characteristics, wealth, and income + health behaviors + prior purpose in life; model 3 is run controlling for sociodemographic characteristics, wealth, and income + health behaviors + health conditions + prior purpose in life; model 4 is run controlling for all covariates + prior purpose in life.

^d^ All missing covariate and outcome variables were imputed using the chained equations. 10 sets of imputed data were generated [58] and the multiple imputation estimates pooled using the Rubin’s rule [59] were presented.

Excluding participants who had any of the health conditions at prebaseline yielded similar results for the standardized AMSEB in all examined models regarding the sets of covariates (Table 4, limited sample). However, for the tertiles of the AMSEB scale, the effects became insignificant and less precise for the second tertile compared to the first tertile in all models. Regarding the third tertile, the effects continued to be significant but were of slightly lower magnitude.

**Table 4:** Robustness analyses. Standardized Regression Estimates for the Association Between Baseline Adherence to Moral Standards and Ethical Behaviors and Purpose in Life over a Four-Year Follow-Up Period in Middle-Aged and Older Adulthood. Health and Retirement Study, US, 2012/2014–2016/2018, Full Case Scenario and Limited Sample Analyses.

Model	Standardized AMSEB		Tertile 1	Tertile 2		Tertile 3	
	*β* (95% CI)	p-value	Reference	*β* (95% CI)	p-value	*β* (95% CI)	p-value
***Limited sample***^***b***^ ***(N = 2*,*405)***							
Model 1^a^	0.059 (0.025; 0.093)	0.001	Reference	0.055 (-0.018; 0.127)	0.142	0.134 (0.03; 0.215)	0.001
Model 2^a^	0.053 (0.020; 0.088)	0.002	Reference	0.041 (-0.033; 0.114)	0.276	0. 123 (0.041; 0.205)	0.003
Model 3^a^	0.050 (0.016; 0.084)	0.004	Reference	0.036 (-0.037; 0.109)	0.336	0.116 (0.034; 0.198)	0.005
Model 5^a^	0.036 (0.003; 0.071)	0.034	Reference	0.021 (-0.052; 0.093)	0.580	0.087 (0.005; 0.169)	0.037
***Full case scenario***							
Model 1^a^ *(N = 8*,*622)*	0.064 (0.045; 0.082)	<0.001	Reference	0.061 (0.002; 0.103)	0.003	0. 167 (0.121; 0.212)	<0.001
Model 2^a^ *(N = 8*,*427)*	0.061 (0.042; 0.080)	<0.001	Reference	0.056 (0.015; 0.099)	0.008	0.160 (0.115; 0.207)	<0.001
Model 3^a^ *(N = 7*,*930)*	0.055 (0.036; 0.074)	<0.001	Reference	0.058 (0.015; 0.104)	0.008	0.156 (0.110; 0.203)	<0.001
Model 4^a^ *(N = 7*,*793)*	0.047 (0.028; 0.066)	<0.001	Reference	0.049 (0.007; 0.092)	0.023	0.141 (0.094; 0.188)	<0.001

AMSEB = adherence to moral standards and ethical behaviors; CI = confidence interval

^a^ Model 1 is run controlling for sociodemographic characteristics, wealth, income + prior purpose in life; model 2 is run controlling for sociodemographic characteristics, wealth, and income + health behaviors + prior purpose in life; model 4 is run controlling for all covariates + prior purpose in life; model 5 is run controlling for sociodemographic characteristics, wealth, and income + health behaviors + psychological factors + prior purpose in life.

^b^ Analysis on a limited sample of people with no health conditions in the pre-baseline wave. Analysis run on the imputed dataset. All missing covariate and outcome variables were imputed using the chained equations. 10 sets of imputed data were generated [58] and the multiple imputation estimates pooled using the Rubin’s rule [59] were presented

With respect to the full case scenario, the significant associations between AMSEB and subsequent purpose in life remained significant in both specifications, i.e., for the standardized indicator of AMSEB and for the tertiles of AMSEB (Table 4, full case scenario). This corroborated the robustness of the associations, with some reservations for people with no health conditions. However, a supplementary analysis with an interaction term between standardized AMSEB and indicator of being free of any health condition yielded no significant estimate for this interaction (S1 Table). This provided further evidence that the prospective association between AMSEB and purpose in life is robust to the health status of respondents.

## Discussion

There has been a growing interest in the role of positive psychological factors and attitudes for health and well-being. Two positive health determinants have attracted academic attention. These are a sense of purpose and/or meaning in life and morally valued personality traits. These two positive factors have been shown to be independently favorably associated with subsequent health and well-being outcomes [11, 30, 61–63]. However, little is known about the antecedents of the two. In this study, we looked for some empirical evidence on whether the adherence to moral standards and ethical behaviors can be perceived as an antecedent of sense of purpose in life. We found that middle-aged and older adults who scored higher on the AMSEB scale (highlighting a predisposition to follow moral standards and behave ethically) after the 4-year follow-up period reported a higher sense of purpose in life. This association was found to be monotonic, moderately robust to potential unmeasured confounding and independent of demographics, prior socioeconomic status, prior health conditions, and health behaviors as well as prior psychological predispositions such as dispositional optimism and life satisfaction.

Our results provide additional evidence on the possible determinants of purpose in life. They show that adherence to moral standards and ethical behaviors can be perceived as a predictor of subsequent purpose in life, which adds to the list of previously identified predictors including social relations, psychological well-being and positive affect, as well psychological distress and mental health conditions already identified in previous studies [17–19, 22, 64]. They also strengthen prior evidence that being a good human being and doing good translate into higher purpose and meaning in life, which was confirmed for one year follow-up [23]. Our results extend this period and show that these associations can be observed even after four years. This, in turn, provides some indications that the adherence to moral standards and ethical behaviors may have the potential to maintain, cultivate and restore purpose in life. Additionally, our results reinforce the evidence from prior studies highlighting that maintaining moral standards and engaging in altruistic activities that contribute to the good of others and themselves may be beneficial for health (e.g., protecting against cognitive impairment, depression, and risky health-related behaviors) and better functioning (e.g., mitigating risks of limitations in mobility and activities of daily living) [30–32]. The implications of our findings are as follows. First, our results indicate a potential contribution of adherence to moral standards and ethical behaviors to greater purpose in life and consequently, to better functioning in middle-aged and older adulthood. Since there is ample evidence that purpose in life decreases in late adulthood [2, 22, 64, 65], the predisposition to follow moral standards and behave ethically seems to be of importance for cultivating purpose in life and indirectly for promoting health and the quality of life in old age. Second, according to self-determination theory, people have intrinsic inclinations toward acting positively and making self-directed efforts to do what is right, moral and needed even in ethically questionable situations [66–68]. Additionally, numerous studies confirm that interventions targeting positive morally valued personality traits are effective [69] and that the traits associated with honesty, integrity and moral values can be stimulated and reinforced over time [25, 70]. Given the above arguments and our results, a predisposition to adhere to moral standards and ethical behaviors has the potential to play a vital role in promoting purpose in life in middle-aged and older adulthood. In other words, having strong moral principles, acting just and doing good for the advancement of one’s and others’ well-being, might be an important, unexploited factor for promoting active and healthy aging.

### Strengths and limitations

This study adds to the literature in the following ways. First, using a large, prospective, and nationally representative sample of US middle-aged and older adults (aged ≥50 years), this study showed a prospective association between the adherence to moral standards and ethical behaviors and purpose in life and thus provided new longitudinal evidence on the probable determinant of purpose in life. Second, the longitudinal design and the adjustment for a wide range of covariates and prior values of the purpose in life outcome provided some support that the established associations are not subject to reverse causation and unmeasured confounding. Third, the sensitivity analysis for unmeasured confounding (using the E-values) provided further evidence for the robustness of identified associations. Finally, a series of secondary analyses strengthened the evidence in favor of the robustness of our results.

Despite its strengths, this study is also subject to certain limitations. In this study, the adherence to moral standards and ethical behaviors was considered an antecedent of purpose in life. It may be, however, that the relation is reciprocal. Due to the design of HRS and lack of repeated measurements of AMSEB in the study, it was not possible, however, to examine the reciprocal relation (i.e., no control for (pre)baseline AMSEB was possible). Future research might follow-up with the examination of bidirectional pathways between purpose in life and AMSEB. Similarly, prevalence rather than incident exposure was evaluated, and it may be a concern. However, this resulted from the study design. Additionally, since personality traits including morally valued ones evolve rather slowly over time [51] and this study did not examine any intervention effect, measuring prevalence of adherence to moral standards and ethical behaviors rather than its incidental change seemed more substantiated.

## Supporting information

S1 Table Standardized regression estimates for the association between baseline adherence to moral standards and ethical behavior and purpose in life over a four-year follow-up period in middle-aged and older adulthood (model with an interaction term). Health And Retirement Study, US, 2012/2014–2016/2018, N = 8,497^a^. AMSEB = adherence to moral standards and ethical behavior; CI = confidence interval; ^a^All missing covariate and outcome variables were imputed using the chained equations. 10 sets of imputed data were generated and the multiple imputation estimates pooled using the Rubin’s rule were presented.
(DOCX)
