# Supplementary material for: Can adherence to moral standards and ethical behaviors help maintain a sense of purpose in life? Evidence from a longitudinal study of middle-aged and older adults
Source: PLoS One. 2022 Aug 19;17(8):e0273221. doi: 10.1371/journal.pone.0273221 (PMC9390941; doi:10.1371/journal.pone.0273221)
Supplement: S1 Table — Health And Retirement Study, US, 2012/2014–2016/2018, N = 8,497a. AMSEB = adherence to moral standards and ethical behavior; CI = confidence interval; aAll missing covariate and outcome variables were imputed using the chained equations. 10 sets of imputed data were generated and the multiple imputation estimates pooled using the Rubin’s rule were presented. (DOCX) [file pone.0273221.s001.docx]

**Can adherence to moral standards and ethical behavior help maintain a sense of purpose in life? Evidence from the longitudinal study of middle-age and older adults**

**SUPPORTING INFORMATION**

**Table S1. Standardized Regression Estimates for the Association Between Baseline Adherence to Moral Standards and Ethical Behavior and Purpose in Life Over a Four-Year Follow-Up Period in Middle-Aged and Older Adulthood (Model with an Interaction Term). Health And Retirement Study, US, 2012/2014 – 2016/2018, N=8,497^a^.**

| **Interaction term** | **Standardized AMSEB** | |
| --- | --- | --- |
|  | $\beta$(95% CI) | p-value |
| AMSEB x gender (ref. = male) | -0.037  (-0.075; 0.002) | 0.065 |
| AMSEB x age group (ref.=50-59) |  |  |
| 60-69 | -0.043  (-0.083; -0.002) | 0.041 |
| 70-79 | 0.012  (-0.036; 0.059) | 0.622 |
| 80+ | -0.009  (-0.092; 0.074) | 0.839 |
| AMSEB x race (ref.= White/Caucasian) |  |  |
| Black/African American | 0.008  (-0.043; 0.059) | 0.753 |
| Other | 0.006  (-0.079; 0.091) | 0.884 |
| AMSEB x education (ref.= Less than high school) |  |  |
| GED | 0.004  (-0.082; 0.089) | 0.935 |
| High school graduate | -0.007  (-0.057; 0.043) | 0.778 |
| Some college | 0.015  (-0.040; 0.070) | 0.594 |
| College and above | -0.000  (-0.056; 0.055) | 0.991 |
| Annual personal income | -0.002  (-0.019; 0.015) | 0.840 |
| Household net financial assets | 0.006  (-0.012, 0.024) | 0.508 |
| AMSEB x being free of any health condition (ref.=no) | 0.002  (-0.037; 0.041) | 0.922 |

﻿ AMSEB= adherence to moral standards and ethical behavior; CI=confidence interval;

^a^All missing covariate and outcome variables were imputed using the chained equations. 10 sets of imputed data were generated and the multiple imputation estimates pooled using the Rubin’s rule were presented.
